# Psychological flexibility and emotional trajectories during a semi‐high‐stakes mathematics exam: Integrating acceptance and commitment therapy with control‐value theory

**DOI:** 10.1111/bjep.70110

**Published:** 2026-07-21

**Authors:** Maryam Zarra‐Nezhad, Teemu Holopainen, Pekka Räsänen, Mikko‐Jussi Laakso, Katarina Alanko

**Affiliations:** ^1^ Turku Research Institute of Learning Analytics, Faculty of Science University of Turku Turku Finland; ^2^ Epilepsia Helsinki, Department of Pediatric Neurology HUS Helsinki University Hospital Helsinki Finland

**Keywords:** academic stress, achievement emotions, adolescents, mathematics exam, psychological flexibility

## Abstract

**Background:**

Exams are a common source of emotions in adolescence, requiring regulation for performance and well‐being. Psychological flexibility (PF), the ability to stay open to difficult thoughts and feelings while pursuing meaningful goals, and psychological inflexibility (PI), involving avoidance and self‐criticism, represent adaptive and maladaptive regulation styles that remain understudied in adolescence.

**Aims:**

We examined whether PF/PI is associated with adolescents' emotional trajectories during a semi‐high‐stakes mathematics examination and whether they differ by gender or mathematics performance.

**Sample:**

Participants were 694 Finnish Year 8–9 students (48.7% girls).

**Methods:**

Before the examination, students completed the Children's Psychological Flexibility Questionnaire (CPFQ) and reported their happiness, relaxation, frustration and boredom across seven phases of the examination. Linear mixed‐effects models estimated emotional trajectories and their associations with PF factors (Committed Action and Awareness; Acceptance and Defusion; Fusion and Self‐Judgement), gender and mathematical performance.

**Results:**

Happiness and relaxation declined during the examination and recovered afterward, whereas frustration and boredom increased. Committed Action and Awareness was associated with greater happiness and phase‐specific variations in frustration, while Acceptance and Defusion were associated with higher relaxation and a stronger late‐exam rebound. Greater PI (i.e. lower Fusion and Self‐Judgement) was linked with lower positive and higher negative emotions. Higher mathematical performance was associated with greater happiness and relaxation, and with lower boredom. A gender interaction showed that boys with high PI reported greater boredom.

**Conclusions:**

Strengthening PF skills, particularly acceptance, defusion and value‐driven action, may help adolescents manage examination stress, sustain engagement and reduce boredom in academic settings.

## INTRODUCTION

Adolescence is marked by heightened emotional intensity, expanding cognitive and social demands, and mounting performance pressures. During this developmental period, approximately one in seven adolescents experiences a mental disorder, accounting for 15% of the global burden of disease in this age group (World Health Organization, n.d.). Academic evaluations, particularly semi‐high‐stakes exams, often elicit emotions that shape motivation and performance (Goetz et al., 2023; Zeidner, 2014).

In the present study, we focused on four core achievement emotions—happiness, relaxation, frustration and boredom—selected to capture students' affective experiences in temporal proximity to engagement with an exam task. These emotions were chosen because they represent commonly studied achievement emotions that differ systematically in valence (positive vs. negative) and activation (activating vs. deactivating), distinctions shown to be central for understanding motivational engagement, persistence and disengagement in academic contexts (Pekrun, 2006; Pekrun & Perry, 2014). Considering emotions across these dimensions allows examination of qualitatively different emotional experiences and their associated coping and self‐regulation patterns during demanding tasks (Pekrun & Perry, 2014).

Emotions that are primarily oriented towards anticipated or future outcomes (e.g. anxiety, hope, hopelessness) were not examined, as our interest was in emotions unfolding in relation to the exam activity itself rather than to results that are expected or received. Although emotions were assessed at different phases surrounding the exam, the measures were intended to capture students' concurrent emotional experiences during the exam context. Importantly, frustration does not represent a unitary arousal state: It may involve high‐arousal components such as irritation or anger, as well as lower‐arousal components such as sadness or resignation, depending on how learners interpret and respond to task‐related obstacles (Pekrun, 2006). Accordingly, the arousal associated with frustration is context‐dependent rather than fixed. Happiness and relaxation support coping and motivation; frustration disrupts persistence, and boredom signals disengagement (Pekrun & Perry, 2014). Because adolescents are still developing the metacognitive and emotional skills needed to manage stress, their ability to regulate exam‐related emotions varies widely. Identifying the mechanisms that help them stay engaged and emotionally balanced under pressure is therefore essential for supporting adaptive adjustment in academic settings.

### Psychological flexibility

A central construct for understanding how adolescents regulate exam‐related emotions is psychological flexibility (PF), the capacity to remain open to difficult thoughts and feelings while acting in ways consistent with personal values (Hayes et al., 2012). In academic settings, students with higher PF can acknowledge exam‐related frustration without being overwhelmed, thereby sustaining focus and engagement despite discomfort. By contrast, psychological inflexibility (PI) involves rigid attempts to avoid or control unwanted experiences, such as suppressing distress or fusing with self‐critical thoughts, which can heighten stress and impair performance (Gillanders et al., 2014; Hayes et al., 2012; Zeidner, 2014).

Rooted in Acceptance and Commitment Therapy (ACT; Hayes et al., 2012), PF comprises six interrelated subprocesses: acceptance (willingness to experience difficult emotions), cognitive defusion (distancing from unhelpful thoughts), present‐moment awareness (attentional flexibility), self‐as‐context (perspective‐taking), values clarification (identifying personally meaningful values and goals) and committed action (persistent value‐consistent behaviour). The Children's Psychological Flexibility Questionnaire (CPFQ; Lenoir et al., 2022) captures these processes specifically in adolescents across three higher‐order dimensions—Committed Action and Awareness (PF‐Action), Acceptance and Defusion (PF‐Accept), and Fusion and Self‐Judgement (PI‐Judge)—providing a developmentally appropriate multidimensional assessment (Langenskiöld et al., 2026). In exam settings, these processes help students tolerate discomfort, defuse self‐critical thoughts and stay focused on valued goals.

PF functions as a meta‐skill supporting emotion regulation and value‐based persistence under pressure. Although related, PF and PI are distinct yet complementary dimensions, each contributing uniquely to well‐being (Cherry et al., 2021; Rolffs et al., 2018). Within this framework, acceptance, defusion and awareness reduce reactivity to distressing thoughts, while values and committed action sustain purposeful engagement.

Empirical evidence indicates that higher PF and lower PI are associated with lower stress and depression symptoms (Puolakanaho et al., 2023), better coping with evaluative stress, including test‐related anxiety (Türk, 2025) and enhanced emotion regulation (Halliburton & Cooper, 2015) in adolescent samples. Comparable patterns have been documented in adult and younger samples across educational and clinical contexts (Bi & Li, 2021; Kashdan & Rottenberg, 2010; López‐Crespo et al., 2025; Macri & Rogge, 2024; Mursalzade et al., 2025; Stabbe et al., 2019; Tyndall et al., 2020). School‐focused syntheses further support the relevance of PF across developmental periods from childhood through adolescence (Blewitt et al., 2024). Beyond correlational findings, increases in PF have been identified as a mechanism of change in ACT‐based and related interventions, mediating treatment‐related reductions in psychological distress, particularly in adult samples (Macri & Rogge, 2024). Importantly, PF appears sensitive to situational demands (Klimczak et al., 2023), underscoring its relevance in performance‐intensive contexts such as examinations.

### Control‐value theory

Control‐Value Theory (CVT; Pekrun, 2006, 2024; Pekrun & Perry, 2014) describes how emotions arise in academic settings. It distinguishes achievement emotions linked to activities (e.g. studying, working on tasks, taking an exam) and those linked to outcomes (e.g. success or failure). In this framework, activity emotions occur during task engagement, whereas outcome emotions stem from anticipated, ongoing or received performance results (Pekrun, 2006, 2024). Activity emotions are shaped by two appraisals: perceived control over the task (e.g. ‘Can I handle this exam?’) and the subjective value attributed to it (e.g. ‘How important is this exam?’). High value paired with low control tends to produce frustration, whereas high control fosters positive emotions such as happiness. CVT further differentiates emotions by object focus (activity vs. outcome) and temporal orientation (prospective, concurrent, retrospective), capturing how emotions unfold before, during and after exams. As perceived control weakens under exam pressure, positive emotions typically decline and recover as students adapt or anticipate task completion, whereas negative emotions increase when control is low or task value diminishes (Pekrun, 2006; Pekrun & Perry, 2014). While CVT specifies the appraisal conditions underlying emotional fluctuations, it does not address the psychological skills that regulate these processes in real time. Integrating PF with CVT therefore allows examination of both the appraisal‐based antecedents and the self‐regulatory processes shaping achievement emotions as they unfold.

Positive and negative emotions can co‐occur rather than exist at opposite ends of a continuum (Cacioppo & Berntson, 1994; Larsen & McGraw, 2011). In mathematics education, emotions such as happiness, relaxation, frustration and boredom often coexist, each showing distinct trajectories and implications for motivation and performance. Evidence comes from both adolescent samples (Gómez et al., 2020; Sakaki et al., 2024) and older student samples (Bieleke et al., 2023). In the present study, however, we examined each emotion separately to model their distinct trajectories across the exam, rather than their co‐occurrence. According to CVT, happiness and relaxation decline as perceived control weakens, frustration rises when goals are blocked and boredom increases with repetitive tasks (Pekrun & Perry, 2014). Longitudinal research in mathematics supports these patterns: happiness and pride decrease across adolescence, and boredom and anger increase, reflecting shifts in control and value appraisals (Sakaki et al., 2024). Together, these findings highlight the importance of assessing multiple emotions to capture valence and arousal dynamics in exam contexts.

### Integrating psychological flexibility with control‐value theory

Integrating PF with CVT offers a comprehensive framework for understanding how students regulate achievement emotions as they unfold. CVT identifies the appraisal pathways that generate these emotions, whereas PF captures the internal processes that support adaptive responses in real time. PF‐Accept can ease the impact of self‐critical thoughts, values clarification enhances task focus, present‐moment awareness anchors attention on the task and committed action maintains effort despite discomfort. Together, these processes illustrate how PF complements CVT by providing mechanisms for in‐the‐moment emotional regulation and sustained engagement during semi‐high‐stakes examinations.

### Developmental and methodological gaps

Early adolescence is a critical period for examining PF, as emotion regulation abilities are still maturing while executive control and self‐reflection are developing but not yet fully efficient (Cole et al., 1994). These capacities underpin ACT subprocesses such as defusion and present‐moment awareness; when underdeveloped, adolescents may be more vulnerable to PI during academic stress (Puolakanaho et al., 2023). As these skills strengthen, students become better able to recognize unhelpful thoughts, tolerate difficult emotions and stay engaged with valued goals (Lenoir et al., 2022). Even those high in trait‐level PF may experience lapses during exams, as effective PF‐Accept depends on momentary cognitive resources, as shown in both adolescent (Farley & Renshaw, 2025) and adult momentary‐assessment studies (Klimczak et al., 2023).

Existing findings support PF as a protective factor across adulthood and adolescence. However, important gaps remain. Most studies rely on cross‐sectional or short‐term designs, limiting inference about temporal dynamics (Cherry et al., 2021; Gloster et al., 2020; Halliburton & Cooper, 2015; Kashdan & Rottenberg, 2010; Liinamaa et al., 2022; Macri & Rogge, 2024; Puolakanaho et al., 2023; Türk, 2025; Wang et al., 2023). In particular, we know little about how PF and PI shape emotional change in evaluatively demanding or potentially stressful situations, or whether these processes operate differently across salient demographic subgroups (e.g. gender).

Methodological constraints also limit current understanding (e.g. Ong et al., 2025). Many common PF measures, such as the Multidimensional Psychological Flexibility Inventory (MPFI; Rolffs et al., 2018), merge flexibility and inflexibility indicators, thereby reducing precision, especially for adolescents. The CPFQ (Lenoir et al., 2022) addresses these gaps with a multidimensional, developmentally appropriate structure that distinguishes PF from PI. Theoretically, while CVT explains how achievement emotions stem from control and value appraisals, it overlooks the self‐regulatory skills that enable students to manage those emotions in real time.

Gender differences warrant explicit consideration in adolescents' emotional responses to evaluative academic contexts. Prior research indicates that girls tend to report higher emotional reactivity in achievement‐related situations, whereas boys are more prone to disengagement and emotional withdrawal under stress or task monotony (Chaplin & Aldao, 2013; Sanchis‐Sanchis et al., 2020). In academic settings, such patterns may translate into gender differences in emotions such as boredom and frustration, particularly when tasks are perceived as demanding or unrewarding. Although PF has been associated with better stress regulation and adjustment in adolescents (Puolakanaho et al., 2023; Türk, 2025), it remains unclear whether PF buffers exam‐related emotions similarly in girls and boys. Examining PF as a potential moderator of gender differences in real‐time emotional trajectories may clarify whether regulation processes operate uniformly across genders or confer differential benefits in evaluative settings.

Together, these developmental, methodological and gender‐related gaps underscore the need for research integrating multidimensional PF assessment with repeated emotion measurements. Such work can clarify how PF and PI shape adolescents' emotional trajectories and whether these processes operate uniformly across genders or exert differential effects during semi‐high‐stakes examinations.

### The present study

In this study, we examined how PF/PI shape adolescents' emotional experiences before, during and after a semi‐high‐stakes mathematics examination, focusing on trajectories of happiness, relaxation, frustration and boredom. This study addresses key gaps identified in prior work, including the limited examination of PF/PI in evaluatively demanding exam contexts in early adolescence, the lack of integration between PF and CVT, the scarcity of repeated in‐exam emotion assessments, ongoing measurement challenges in distinguishing flexibility from inflexibility and unclear gender‐related differences in how PF relates to exam emotions. Building on the empirically validated three‐factor structure of the CPFQ—PF‐Action, PF‐Accept and PI‐Judge (Langenskiöld et al., 2026)—we tested whether these domains were associated with distinct emotional trajectories across the seven exam phases (T1–T7). Items within the PI‐Judge factor were reverse‐scored so that lower scores indicated greater PI. This design allowed us to model within‐exam emotional change and integrate PF's process‐based framework with CVT's account of achievement emotions. Following CVT, happiness and relaxation were expected to decline, and frustration and boredom to rise, providing baseline trajectories for testing PF, gender and mathematics performance effects. We addressed three research questions (RQs):

RQ1) Does PF relate to adolescents' emotion trajectories during the exam? Higher PF‐Action and PF‐Accept were expected to be associated with more stable or rebounding happiness and relaxation, and lower frustration and boredom, consistent with ACT's emphasis on goal‐directed persistence and acceptance under stress, and prior evidence linking acceptance and mindfulness to better emotion regulation in adolescents (Gillanders et al., 2014; Halliburton & Cooper, 2015; Puolakanaho et al., 2023; Türk, 2025). Conversely, lower PI‐Judge (reflecting greater PI) was expected to be associated with higher negative and lower positive emotions (Liinamaa et al., 2022).
RQ2) Does PF moderate gender differences in exam emotions? Girls generally report greater evaluative stress and emotional reactivity than boys (Chaplin & Aldao, 2013; Sanchis‐Sanchis et al., 2020). Emerging evidence suggests that PF may attenuate such patterns by reducing rigid avoidance and supporting adaptive regulation (Puolakanaho et al., 2023; Türk, 2025). However, it remains unclear whether PF moderates gender differences in real‐time exam emotions; therefore, this question was examined exploratorily, without directional hypotheses.
RQ3) Does mathematical performance moderate PF–emotion associations? Given that academic competence shapes control‐value appraisals (Sakaki et al., 2024), PF may be particularly beneficial under higher academic demands (Puolakanaho et al., 2023; Türk, 2025). However, empirical evidence remains limited; therefore, this question was examined exploratorily.

## METHODS

### Participants and procedure

Data were collected in April 2025 as part of the DigiEva project, a large‐scale assessment of mathematical skills conducted across grades 3, 6, 8 and 9 in Finnish‐ and Swedish‐speaking schools in Finland (Metsämuuronen & Laakso, 2025). The full dataset comprised 3362 students.

DigiEva is a curriculum‐based mathematics exam administered at the end of the school year. Students were informed that their performance would contribute to their final mathematics grade, making the exam a semi‐high‐stakes situation likely to elicit stress and emotion regulation demands. Students were informed that their performance would contribute to their final mathematics grade, although the exact weighting varied by teacher.

The present study focused on Finnish‐speaking eighth‐ and ninth‐grade students who completed both the mathematics exam and socio‐emotional measures. After excluding participants with incomplete data or implausible response times, the final analytic sample comprised 694 students (48.7% girls; 45.7% eighth and 54.3% ninth graders), aged 14–16 years. Assessments were administered electronically via the ViLLE digital learning platform (Laakso et al., 2018). At baseline, students reported PF. Emotional experiences were subsequently measured at seven time points across the exam period: once before the exam (T1), five times during the two‐hour exam (T2–T6) and once immediately after the exam (T7). At each measurement point, students reported their current levels of happiness, relaxation, frustration and boredom on a five‐point Likert scale.

The study complied with Finnish law and adhered to the ethical principles for research with human participants issued by the Finnish National Board on Research Integrity (TENK, 2023) and the Declaration of Helsinki. As it involved standard school assessments, no formal ethics review was required. Research permissions were obtained from the participating municipalities. Guardians were informed about the study, and students participated voluntarily under a passive consent procedure with the option to decline participation. All data were pseudonymized before analysis, and no identifying information is available to researchers.

### Measures

#### Psychological flexibility/inflexibility

PF/PI was assessed at baseline (T1) using a shortened nine‐item version of the CPFQ (Langenskiöld et al., 2026), which measures three factors with three items each: PF‐Action (e.g. ‘I try really hard every day’), PF‐Accept (e.g. ‘It's OK to be scared’) and PI‐Judge (e.g. ‘If I get angry, it means I messed up’). The first two domains represent PF and the third PI, reflecting their related but distinct nature. While this structure overlaps conceptually with the youth ‘triflex’ model (open, aware, active; Harris, 2019), the correspondence is not exact. The ‘open’ component broadly aligns with PF‐Accept, whereas ‘aware’ and ‘active’ correspond partly to PF‐Action. Unlike the triflex, which views inflexibility as the absence of flexibility, the CPFQ follows multidimensional approaches that treat PF and PI as asymmetrical but correlated constructs (Ong et al., 2025; Rolffs et al., 2018). Langenskiöld et al. (2026) supported the CPFQ three‐factor structure using a cross‐validation design in which eighth and ninth graders were split for EFA and CFA, with the validation sample supplemented by 6th graders. They reported partial scalar invariance across age and gender and handled missing data using FIML.

In the present study, we tested the same three‐factor model in our full sample of eighth and ninth graders using CFA (Table S1) and replicated the overall structure (Table S2). Internal consistency was acceptable given the short subscales (PF‐Action *α* = .70; PF‐Accept *α* = .67; PI‐Judge *α* = .65; Cortina, 1993; Loewenthal & Lewis, 2018). Gender invariance testing supported partial scalar invariance after freeing three intercepts, one for each factor (Table S3). Missing data were addressed using multiple imputation, and factor scores were derived from the partially invariant model and averaged across imputations.

#### Emotion ratings

Emotion ratings were assessed using single‐item measures to minimize participant burden and maximize compliance in the intensive repeated‐measures design. Although practical, single‐item indicators may offer lower reliability, less nuance and greater susceptibility to measurement error than multi‐item scales.

Emotions were measured at seven time points during a semi‐high‐stakes mathematics exam: once before (T1), five times during (T2–T6) and once immediately after (T7). In‐exam ratings were administered following completion of each designated item cluster. Although minor variation in item order was possible, students generally completed the exam linearly, making these time points a valid representation of emotional changes across the task. At each point, they rated happiness, relaxation, frustration and boredom in response to the question ‘How do you feel?’ on a five‐point scale (1 = not at all to 5 = very much). The emotion ratings were brief and minimally directive; therefore, the items did not specify the exam activity or a time frame (e.g. ‘right now’); the assessed emotions cannot be assumed to be exclusively activity‐focused. These emotions were selected to capture differences in both valence and arousal, consistent with the CVT of achievement emotions: happiness (positive, high arousal), relaxation (positive, low arousal), frustration (negative, commonly associated with higher arousal) and boredom (negative, low arousal). Repeated measurement enabled modelling of within‐person emotional trajectories (e.g. rising frustration or post‐exam recovery).

#### Covariates

Models controlled for students' mathematics test score (continuous), gender (boy = 0, girl = 1) and time (measurement occasion). The total test score was used as the proficiency covariate, providing a global index of mathematical skill rather than relying on a single subscore. Trial‐level performance data preceding each emotion rating were unavailable. Time was modelled as a continuous variable spanning the seven measurement points (T1 pre‐exam; T2–T6 during; T7 post‐exam), with both linear and quadratic trends tested. All predictors except gender were standardized (*M* = 0, SD = 1) before modelling.

### Analyses

Factor scores for the three CPFQ domains (PF‐Action, PF‐Accept, PI‐Judge), derived from the partially invariant measurement model, were used in all analyses to ensure valid gender comparisons. Associations between baseline PF and emotional trajectories across T1–T7 were examined using linear mixed‐effects models implemented in R with the lme4 package (Bates et al., 2015) and lmerTest (Kuznetsova et al., 2017). All fitted models converged successfully. Emotion ratings were treated as continuous, consistent with evidence supporting parametric analyses of ordinal data (Norman, 2010; Sullivan & Artino Jr., 2013).

Model building began by evaluating the necessity of random slopes for time against random‐intercepts‐only models using likelihood ratio tests (Table S1A–D). Next, full models including all PF/PI factors and their theoretically plausible interactions with covariates (time, gender, mathematics score) were fit. To assess potential non‐linearity, time was decomposed into linear and quadratic polynomial terms, which were included in both main effects and interaction terms with the PF/PI factors. Initial checks (visual inspection and statistical tests) showed no evidence of cubic time trends.

The full models were used to identify all potentially meaningful interaction terms and to establish parsimonious base models for further refinement. Subsequent model selection among competing structures was conducted on these base models using likelihood ratio tests (ANOVA), limiting the number of formal model comparisons and reducing the risk of capitalizing on chance (Table S2A–D). In cases of borderline likelihood ratio test results, AIC and BIC values were considered in selection decisions to balance model fit against complexity.

Throughout refinement, the theoretical hierarchy was maintained by retaining (1) main effects involved in significant interactions, (2) time parameters to preserve the longitudinal structure and (3) demographic covariates regardless of significance. Model comparisons were conducted using maximum likelihood estimation, whereas the final models were refit using Restricted Maximum Likelihood to obtain unbiased variance component estimates. Post‐hoc *p*‐values were adjusted using the Benjamini–Hochberg false discovery rate correction (Table S3A–D). Missing repeated‐measures data were assumed missing at random, which mixed‐effects estimation accommodates (Molenberghs & Verbeke, 2000).

## RESULTS

### Preliminary analyses

Prior to the main analyses, data quality and descriptive characteristics were examined. Of the nine CPFQ items, complete responses were available for 643 students (92.7%), with item‐level missingness ranging from .3% to 2.4%. Emotion ratings were scheduled at seven measurement points (T1–T7) and were available for up to 694 students per time point. Complete emotion data across all time points were available for 413 students (59.5%), with missingness peaking at T6 (for relaxation and frustration, 238 missing ratings, 34.3%). Sensitivity analyses excluding T6 confirmed the robustness of the findings. Descriptive statistics for PF/PI factors, mathematics score and emotion ratings across T1–T7 are presented in Table 1. Average correlations among PF/PI factors, mathematics performance and emotions across the seven measurement occasions are shown in Table 2. The adaptive PF domains, PF‐Action and PF‐Accept, were moderately correlated (*r* = .58), while correlations with PI‐Judge were small (*r* = −.13 with PF‐Accept; *r* = .12 with PF‐Action). Variance inflation factors (1.07–1.60) indicated no multicollinearity concerns.

**TABLE 1:** Descriptive statistics of numeric variables.

Variable	*n*	Mean	SD	Median	Trimmed	MAD	Min	Max	Range	Skew	Kurtosis	SE
PF‐Accept	694	−0.10	0.74	−0.02	−0.08	0.79	−2.73	1.32	4.05	−0.38	−0.06	0.03
PF‐Action	694	−0.02	0.31	0.00	0.00	0.29	−1.33	0.53	1.86	−0.75	0.86	0.01
PI‐Judge	694	0.16	0.49	0.23	0.19	0.45	−1.24	1.06	2.30	−0.53	0.07	0.02
T10 (math score)	689	−0.00	1.00	−0.01	−0.01	0.98	−2.70	3.23	5.93	0.11	−0.04	0.04
Bored_T1	690	3.25	1.31	3.00	3.32	1.48	1.00	5.00	4.00	−0.23	−1.08	0.05
Bored_T2	691	3.35	1.42	4.00	3.44	1.48	1.00	5.00	4.00	−0.29	−1.27	0.05
Bored_T3	681	3.44	1.47	4.00	3.55	1.48	1.00	5.00	4.00	−0.38	−1.28	0.06
Bored_T4	671	3.50	1.51	4.00	3.62	1.48	1.00	5.00	4.00	−0.46	−1.29	0.06
Bored_T5	661	3.56	1.54	4.00	3.71	1.48	1.00	5.00	4.00	−0.54	−1.27	0.06
Bored_T6	459	3.55	1.55	4.00	3.69	1.48	1.00	5.00	4.00	−0.53	−1.28	0.07
Bored_T7	635	3.52	1.51	4.00	3.65	1.48	1.00	5.00	4.00	−0.48	−1.26	0.06
Frustrated_T1	690	2.26	1.34	2.00	2.07	1.48	1.00	5.00	4.00	0.76	−0.64	0.05
Frustrated_T2	684	2.21	1.33	2.00	2.02	1.48	1.00	5.00	4.00	0.81	−0.56	0.05
Frustrated_T3	679	2.34	1.44	2.00	2.18	1.48	1.00	5.00	4.00	0.73	−0.85	0.06
Frustrated_T4	665	2.37	1.48	2.00	2.22	1.48	1.00	5.00	4.00	0.66	−1.00	0.06
Frustrated_T5	657	2.49	1.53	2.00	2.37	1.48	1.00	5.00	4.00	0.53	−1.22	0.06
Frustrated_T6	456	2.44	1.50	2.00	2.31	1.48	1.00	5.00	4.00	0.62	−1.09	0.07
Frustrated_T7	634	2.47	1.51	2.00	2.34	1.48	1.00	5.00	4.00	0.56	−1.15	0.06
Happy_T1	693	2.46	1.10	2.00	2.41	1.48	1.00	5.00	4.00	0.25	−0.76	0.04
Happy_T2	688	2.12	1.08	2.00	1.98	1.48	1.00	5.00	4.00	0.78	0.00	0.04
Happy_T3	679	1.94	1.06	2.00	1.77	1.48	1.00	5.00	4.00	0.96	0.10	0.04
Happy_T4	669	1.83	1.05	1.00	1.65	0.00	1.00	5.00	4.00	1.15	0.51	0.04
Happy_T5	658	1.75	1.03	1.00	1.57	0.00	1.00	5.00	4.00	1.24	0.64	0.04
Happy_T6	457	1.75	1.02	1.00	1.57	0.00	1.00	5.00	4.00	1.23	0.68	0.05
Happy_T7	638	1.83	1.10	1.00	1.63	0.00	1.00	5.00	4.00	1.15	0.31	0.04
Relaxed_T1	692	2.55	1.18	2.00	2.49	1.48	1.00	5.00	4.00	0.28	−0.91	0.05
Relaxed_T2	688	2.31	1.15	2.00	2.22	1.48	1.00	5.00	4.00	0.49	−0.67	0.04
Relaxed_T3	680	2.15	1.15	2.00	2.03	1.48	1.00	5.00	4.00	0.66	−0.58	0.04
Relaxed_T4	667	2.05	1.14	2.00	1.89	1.48	1.00	5.00	4.00	0.83	−0.31	0.04
Relaxed_T5	655	1.94	1.13	1.00	1.76	0.00	1.00	5.00	4.00	0.93	−0.18	0.04
Relaxed_T6	456	1.91	1.12	1.00	1.73	0.00	1.00	5.00	4.00	0.99	−0.05	0.05
Relaxed_T7	636	1.97	1.14	2.00	1.80	1.48	1.00	5.00	4.00	0.93	−0.13	0.05

Abbreviations: Kurtosis, bias corrected kurtosis; MAD, median absolute deviation (from the median); *n*, number of valid cases; PF‐Accept, Acceptance and Defusion; PF‐Action, Committed Action and Awareness; PI‐Judge, Fusion and Self‐Judgement; SD, standard deviation; SE, standard error of the mean; Skew, bias corrected skewness; Trimmed, trimmed mean (with trim defaulting to .1).

**TABLE 2:** Average correlation matrix across seven time points using fisher's Z‐transformed correlations.

Variable	PF‐accept	PF‐action	PI‐judge	Bored	Frustrated	Happy	Relaxed	T10
PF‐Accept		.58	−.13	−.07	−.04	.13	.09	.19
PF‐Action	.58		.12	−.13	−.08	.25	.20	.24
PI‐Judge	−.13	.12		−.02	−.18	.15	.23	.07
Bored	−.07	−.13	−.02		.30	−.32	−.18	−.13
Frustrated	−.04	−.08	−.18	.30		−.26	−.24	−.03
Happy	.13	.25	.15	−.32	−.26		.66	.16
Relaxed	.09	.20	.23	−.18	−.24	.66		.19
T10 (math score)	.19	.24	.07	−.13	−.03	.16	.19	

*Note*: Correlations among the four emotion variables were computed separately for each of the seven measurement occasions and then Fisher *z*‐transformed and averaged to yield a single cross‐time estimate of their associations. Psychological flexibility factors and math score (T10) were treated as time‐invariant variables and were correlated with the averaged emotion scores. All values represent Pearson's correlations based on pairwise complete data.

Abbreviations: PF‐Accept, Acceptance and Defusion; PF‐Action, Committed Action and Awareness; PI‐Judge, Fusion and Self‐Judgement.

### Emotion trajectories

We examined whether the three CPFQ factors—PF‐Action, PF‐Accept and PI‐Judge—were associated with emotion trajectories across the exam. Linear mixed‐effects models tested associations between PF/PI and emotions over time. Given limited prior evidence on which PF/PI factors influence emotions, we employed the model selection strategy described in the Methods section to identify the most important predictors for each emotion. Final fixed‐effect estimates and interactions are summarized in Table 3, and Figures 1 and 2 show the modelled trajectories for positive and negative emotions. Random effects and model fit statistics are reported in the Supporting Information (Table S4A–D).

**TABLE 3:** Fixed‐effect estimates for predictors and interactions in the four modelled emotions.

Emotions	Predictor	Estimate	95% CI	Adjusted *p*
Happiness	(Intercept)	1.94	1.83–2.04	**<.001**
	gender [girl]	−.30	−.43 to −.17	**<.001**
	time linear	−.21	−.23 to −.19	**<.001**
	time quadratic	.15	.13–.17	**<.001**
	PF‐Action	.77	.55–.99	**<.001**
	T10 (math score)	.10	.04–.17	.**003**
	PI‐Judge	.15	.02–.30	.**025**
	time quadratic × PF‐Action	.06	.00–.12	.**036**
Relaxation	(Intercept)	2.11	2.01–2.22	**<.001**
	PI‐Judge	.44	.29–.58	**<.001**
	time linear	−.20	−.23 to −.17	**<.001**
	gender [girl]	−.33	−.47 to −.19	**<.001**
	time quadratic	.11	.09–.13	**<.001**
	PF‐Accept	.13	.03–.23	.**010**
	T10 (math score)	.18	.11–.25	**<.001**
	time quadratic × PF‐Accept	.03	.01–.06	.**019**
Frustration	(Intercept)	2.51	2.37–2.65	**<.001**
	time linear	.10	.07–.13	**<.001**
	PF‐Action	−.02	−.40 to .36	.911
	time quadratic	−.01	−.03 to .02	.662
	PI‐Judge	−.55	−.75 to −.36	**<.001**
	gender [girl]	−.09	−.27 to .10	.494
	T10 (math score)	−.02	−.11–.07	.751
	PF‐Accept	−.09	−.24 to .07	.426
	time linear × PF‐Action	.14	.03–.25	.**022**
	time quadratic × PF‐Action	−.19	−.30 to −.09	.**001**
	time quadratic × PF‐Accept	.06	.02–.10	.**015**
Boredom	(Intercept)	3.74	3.59–3.90	**<.001**
	time linear	.10	.06–.14	**<.001**
	time quadratic	−.06	−.09 to −.03	**<.001**
	T10 (math score)	−.18	−.27 to −.10	**<.001**
	gender [girl]	−.33	−.52 to −.13	.**002**
	PI‐Judge	−.41	−.70 to −.11	.**008**
	gender [girl] × PI‐Judge	.43	.05–.81	.**028**

*Note*: Adjusted *p* = Benjamini–Hochberg adjusted *p*‐ value; statistically significant *p*‐ values are in bold. All predictors except gender were scaled before model fitting. Models include random effects in addition to the reported fixed effects.

Abbreviations: CI, 95% confidence interval; PF‐Accept, Acceptance and Defusion; PF‐Action, Committed Action and Awareness; PI‐Judge, Fusion and Self‐Judgement.

**FIGURE 1:**
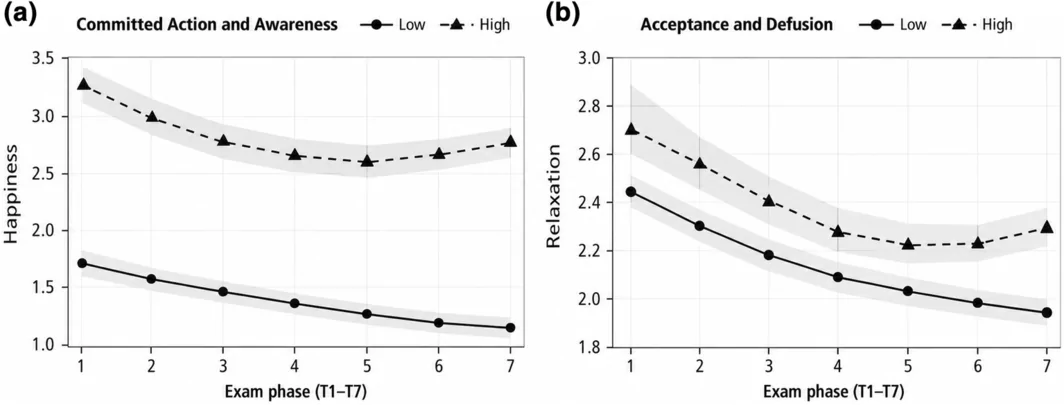
Predicted trajectories of positive emotions across the seven exam phases (T1–T7) as a function of psychological flexibility processes. (a) Happiness by Committed Action and Awareness and (b) relaxation by Acceptance and Defusion. Lines represent modelled emotion levels for adolescents low (−1 SD) and high (+1 SD) in each Psychological Flexibility factor, derived from linear mixed‐effects models. Shaded areas show 95% confidence intervals for predictions. The *Y*‐axis is truncated to highlight changes in emotional trajectories. Higher Committed Action and Awareness and Acceptance and Defusion were associated with greater positive emotion and faster emotional recovery during the exam.

**FIGURE 2:**
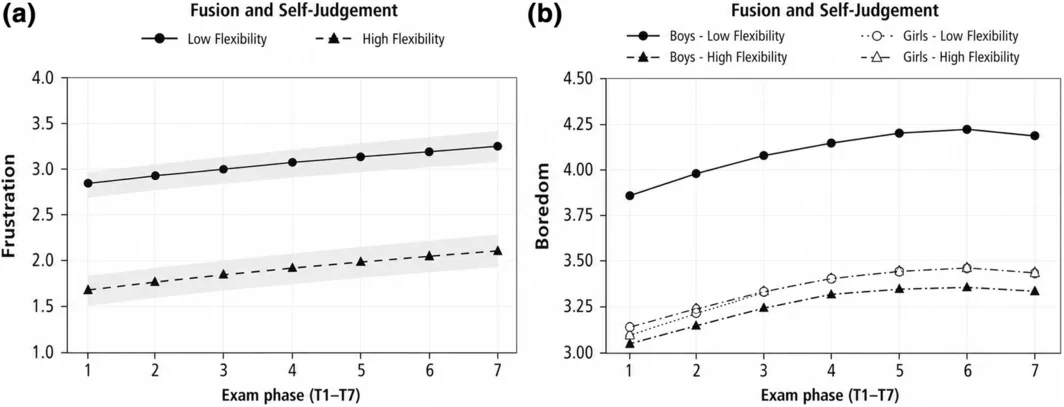
Predicted trajectories of negative emotions across the seven exam phases (T1–T7) as a function of psychological flexibility and inflexibility. (a) Frustration by Fusion and Self‐Judgement and (b) Boredom by gender × Fusion and Self‐Judgement. Lines represent modelled emotion levels for adolescents low (−1 SD; high inflexibility) and high (+1 SD; high flexibility) in each process, estimated from linear mixed‐effects models. Shaded areas show 95% confidence intervals for predictions. The *Y*‐axis is truncated to highlight changes in emotional trajectories. Greater psychological flexibility was associated with less negative emotion and more stable trajectories across the exam.

#### Happiness

Happiness showed a U‐shaped trajectory across the exam period, declining initially and then partially rebounding (linear *β* = −.21, *p* < .001; quadratic *β* = .15, *p* < .001). Higher mathematics performance was slightly associated with greater happiness (*β* = .10, *p* = .003) and lower PI‐Judge scores (reflecting greater PI) with lower happiness (*β* = .15, *p* = .025). Girls reported moderately less happiness than boys (*β* = −.30, *p* < .001). The quadratic time × PF‐Action interaction was significant in the primary model (*β* = .06, *p* = .036) but not in the sensitivity analysis excluding T6 (*β* = .05, *p* = .148), suggesting a small, unstable effect. The main effect of PF‐Action indicated a robust and substantial association with baseline happiness (*β* = .77, *p* < .001). The model explained 15.7% of the variance through fixed effects (marginal *R* ^2^) and 76.6% when both fixed and random effects were considered (conditional *R* ^2^) (Figure 1a).

#### Relaxation

Relaxation showed a U‐shaped pattern across the exam, declining initially and then partially rebounding (linear *β* = −.20, *p* < .001; quadratic *β* = .11, *p* < .001). Lower PI‐Judge scores (reflecting greater PI) were substantially associated with lower relaxation (*β* = .46, *p* < .001). Girls reported substantially less relaxation than boys (*β* = −.33, *p* < .001), and higher mathematics performance (*β* = .18, *p* < .001) was modestly associated with greater relaxation. A small but significant quadratic time × PF‐Accept interaction (*β* = .03, *p* = .019) indicated that greater PF was associated with a stronger rebound in relaxation later in the exam. The model explained 14.1% of the variance from fixed effects alone (marginal *R* ^2^) and 71.7% when including random effects (conditional *R* ^2^) (see Figure 1b).

#### Frustration

Frustration increased modestly over time (*β* = .10, *p* < .001). Lower PI‐Judge scores (reflecting greater PI) were substantially associated with higher frustration (*β* = −.55, *p* < .001), independent of gender and mathematics performance (see Figure 2a). While the quadratic main effect of time was not significant, small to modest interactions indicated that PF effects varied across exam phases: quadratic time × PF‐Accept (*β* = .06, *p* = .015), quadratic time × PF‐Action (*β* = −.19, *p* = .001) and linear time × PF‐Action (*β* = .14, *p* = .022). The model explained 4.4% of the variance through fixed effects (marginal *R* ^2^) and 71.8% when both fixed and random effects were considered (conditional *R* ^2^).

#### Boredom

Boredom increased slightly over time with a decelerating pattern (linear *β* = .10, *p* < .001; quadratic *β* = −.06, *p* < .001). Higher mathematics performance was modestly associated with less boredom (*β* = −.18, *p* < .001). The interaction between gender and PI‐Judge was significant (*β* = .43, *p* = .029), indicating that the effect of PI on boredom differed by gender. Lower PI‐Judge scores (reflecting greater PI) were substantially associated with higher boredom for boys. The model explained 3% of the variance from fixed effects alone (marginal *R* ^2^) and 68.4% when including random effects (conditional *R* ^2^) (see Figure 2b).

## DISCUSSION

This study examined how PF/PI relates to adolescents' emotions before, during and after a semi‐high‐stakes mathematics exam. Happiness and relaxation followed U‐shaped trajectories, declining during the exam and rebounding afterwards, whereas frustration and boredom increased. PF‐Action was associated with higher happiness and phase‐specific increases in frustration, PF‐Accept with greater relaxation and a stronger late‐exam rebound, and greater PI (lower PI‐Judge) with lower happiness and relaxation, but with higher frustration and boredom. Higher mathematics performance was associated with greater happiness and relaxation and with less boredom, whereas greater PI was linked to higher boredom among boys only. Given the modest internal consistency of our shortened CPFQ subscales, particularly the inflexibility component, we emphasize practical significance and effect sizes over statistical significance alone. Smaller effects are interpreted cautiously and considered tentative.

### Happiness

Happiness followed a U‐shaped trajectory, consistent with CVT's account that positive achievement emotions often decline during demanding periods and recover as students approach task completion. Because control and value appraisals were not measured in this study, the links to CVT should be interpreted as theoretical inferences rather than direct tests of appraisal mechanisms. Consistent with RQ1, PF‐Action was associated with greater happiness, suggesting that value‐based persistence may support engagement and well‐being under pressure. This is consistent with ACT's emphasis on goal‐directed behaviour amid discomfort and evidence linking PF to emotional recovery during academic stress (Puolakanaho et al., 2023; Türk, 2025). However, the time × PF‐Action interaction was small and unstable across sensitivity analyses, providing only tentative evidence that PF‐Action meaningfully shaped the trajectory of happiness during the exam.

Greater PI (lower PI‐Judge) was associated with lower happiness, consistent with prior findings that fusion and self‐criticism heighten distress (Gillanders et al., 2014; Gloster et al., 2020). The capacity to notice rather than over‐identify with self‐critical thoughts may help adolescents preserve a sense of control and facilitate more rapid emotional recovery. Overall, these findings suggest small but consistent associations, indicating that behavioural engagement (committed action) and cognitive openness (defusion or low self‐judgement) foster positive affect in evaluative settings. PF effects did not vary by gender (RQ2), although girls reported lower overall happiness, consistent with previous evidence of stronger evaluative stress in girls (Chaplin & Aldao, 2013; Sanchis‐Sanchis et al., 2020). Higher mathematics performance was associated with greater happiness (RQ3), consistent with the positive links among perceived competence, control and enjoyment in achievement contexts (Pekrun & Perry, 2014; Sakaki et al., 2024).

### Relaxation

Relaxation also followed a U‐shaped trajectory, declining early in the exam and rebounding towards its end. This pattern aligns with CVT, which suggests that perceived control weakens during demanding phases but recovers as students adapt or anticipate completion. Consistent with RQ1, PF‐Accept was associated with higher overall relaxation and a slightly stronger late‐exam rebound, suggesting that adolescents who can accept discomfort and defuse from self‐critical thoughts may experience smoother recovery of calm once task demands stabilize. Within the ACT framework, acceptance reduces struggles with uncomfortable internal experiences, while defusion allows individuals to observe thoughts without reacting to them as literal truths, thereby reducing tension and facilitating recovery. Empirical evidence similarly links mindfulness and acceptance processes to lower stress and improved regulation in adolescents (Gillanders et al., 2014; Halliburton & Cooper, 2015).

A greater PI (lower PI‐Judge) was associated with lower relaxation, consistent with evidence that cognitive fusion and self‐criticism heighten physiological and emotional reactivity under stress (Gloster et al., 2020; Puolakanaho et al., 2023). Adolescents who are less entangled in self‐critical thoughts are more likely to interpret exam stress as temporary rather than self‐defining, enabling smoother downregulation of arousal. These findings suggest modest, complementary roles of PF‐Accept and of low Fusion/Self‐Judgement in supporting composure and emotional stability under evaluative pressure. No significant moderation by gender or mathematics performance was observed (RQ2–RQ3), indicating that these associations were broadly consistent across students.

### Frustration

Frustration increased modestly across exam phases, a pattern consistent with CVT, which proposes that negative activating emotions intensify when valued goals are obstructed and perceived control declines. Consistent with RQ1, greater PI (lower PI‐Judge) was associated with higher overall frustration, supporting evidence that self‐critical and fused thinking heighten stress reactivity under evaluative challenges (Gillanders et al., 2014; Puolakanaho et al., 2023). Beyond these main effects, frustration patterns showed small variations associated with PF components. Students high in PF‐Action showed a slight increase in frustration early in the exam, followed by a modest decline towards the later stages. By contrast, students higher in PF‐Accept exhibited a smaller increase in frustration during the later phase of the exam. Given the small magnitude of these interaction effects, these patterns should be interpreted cautiously and viewed as preliminary indications rather than strong evidence of PF‐driven changes in emotional trajectories.

Based on the ACT framework, value‐based engagement and openness to internal experiences may help individuals adjust to discomfort rather than react rigidly to it, offering a conceptual explanation for these modest associations. Rather than suppressing frustration, adolescents with higher PF may be somewhat more able to allow and adapt to it, maintaining goal‐directed behaviour despite discomfort. This interpretation is supported by empirical evidence linking PF processes to lower stress, better coping and fewer negative emotions in academic contexts (Puolakanaho et al., 2023; Türk, 2025). No significant moderation effects were found for gender or mathematics performance (RQ2–RQ3), indicating that the small PF‐related associations with frustration operated similarly across students.

### Boredom

Boredom increased slightly across exam phases, consistent with CVT, which posits that boredom arises when tasks are perceived as monotonous, overly demanding or lacking personal value. Consistent with RQ1, greater PI (lower PI‐Judge) was associated with higher boredom, suggesting that self‐critical and fused thinking undermines sustained engagement as motivation declines. From an ACT perspective, cognitive defusion and reduced self‐judgement can help individuals remain present and task‐focused rather than disengaging in avoidance. Adolescents higher in PF may therefore be somewhat better able to sustain attention and effort even when tasks feel tedious or challenging, although the effect observed here was modest. Empirical findings similarly show that PF is associated with attention, persistence and emotion regulation in academic settings (Puolakanaho et al., 2023; Türk, 2025).

Consistent with RQ2, a significant gender interaction indicated that the association between PF and boredom was stronger for boys, who appeared more prone to disengagement when inflexibility was high. This pattern may reflect gendered coping differences: Girls typically report greater emotional awareness and use of regulatory strategies (Chaplin & Aldao, 2013; Sanchis‐Sanchis et al., 2020), whereas boys are more susceptible to behavioural withdrawal when tasks become stressful or unengaging. Although exploratory, this pattern provides a preliminary indication that PF processes may be more relevant for boys in situations where inflexibility is high, but stronger evidence is needed before drawing applied conclusions. This interpretation aligns with prior work suggesting that PF can buffer emotional reactivity and may help reduce gender differences in stress responses (Türk, 2025). Mathematics performance did not moderate these associations (RQ3), indicating that PF buffered boredom consistently across ability levels.

### Strengths and limitations

This study integrates the ACT model of PF with CVT, linking control‐value appraisals with regulatory processes that support emotional balance during examinations. Focusing on early adolescence captures PF at a developmental stage characterized by high stress reactivity and still‐maturing regulatory skills. The seven repeated emotion measurements provide a detailed picture of within‐exam emotional change, improving on cross‐sectional or two‐wave designs. The use of the multidimensional CPFQ allows PF and PI to be distinguished, and a large, ecologically valid sample of Finnish eighth‐ and ninth‐grade students strengthens the study's statistical power and relevance to real exam contexts.

Several limitations should be considered when interpreting the present findings. First, some emotion ratings, particularly at T6, were missing; however, sensitivity analyses suggested no impact on the study's conclusions. In addition, although the shortened CPFQ reduced participant burden, the PI‐Judge subscale demonstrated modest reliability, highlighting the need for continued refinement of psychological flexibility measures for adolescents. Second, the absence of trial‐level performance data limited the examination of moment‐to‐moment links between psychological flexibility, performance and emotions; future research integrating fine‐grained performance indicators with repeated emotion sampling could address this gap. Third, reliance on self‐reported emotions entails subjective interpretation and shared‐method variance. Moreover, although the emotions were theoretically situated within CVT, ratings collected before, during and after the exam may not have been interpreted consistently as activity‐focused achievement emotions by all students, particularly during phases close to anticipated or perceived outcomes. Fourth, repeated in‐exam assessments—conducted multiple times during the exam and once before and after—may have influenced emotional experiences by interrupting immersion or increasing frustration levels, and potential assessment reactivity should therefore be considered when interpreting the findings. Fifth, gender was assessed using a binary response format, which does not capture the full spectrum of gender identities and may have affected the validity of gender‐related findings due to potential misclassification or exclusion of gender‐diverse participants (Cameron & Stinson, 2019). Finally, the generalizability of the results may be limited to the national context examined, as exam practices and perceived stakes vary across educational systems and cultures.

### Implications for educational psychology

Our findings highlight PF as a central self‐regulatory skill for managing evaluative stress in adolescence. Integrating ACT with CVT clarifies how acceptance, defusion and value‐based persistence support emotional balance when control appraisals weaken. In practice, PF training can be embedded into social–emotional learning or test‐preparation programmes to help students tolerate discomfort, sustain engagement and reduce boredom during academically demanding tasks. Strengthening these skills may support well‐being and academic persistence, offering a practical route for educators and school psychologists to foster resilience under pressure.

### Future research directions

Future research should investigate PF's moment‐to‐moment role using real‐time performance data and control‐value appraisals. Adaptive assessments that calibrate task difficulty to skill level may provide more comparable emotional contexts and clarify how PF operates under conditions of balanced demands. Multi‐method designs incorporating experience sampling, physiological indicators and objective performance measures would further elucidate flexible regulation during academic tasks. Continued validation of adolescent‐specific PF measures, replication across diverse educational settings and longitudinal and intervention studies are needed to strengthen generalizability and test whether PF training causally enhances emotion regulation and academic achievement.

## CONCLUSION

This study examined adolescents' emotions before, during and after a mathematics examination, and their associations with mathematical performance, gender and the ability to manage difficult thoughts and feelings. Higher mathematical performance was associated with greater happiness and relaxation, and with lower levels of boredom. PF showed a protective role, particularly for boys, who were more prone to disengagement under high inflexibility. Adolescents able to accept difficult internal experiences while pursuing valued goals showed more effective emotion regulation, recovering from frustration and remaining calm as the exam progressed. By contrast, greater inflexibility, marked by self‐critical or avoidant thinking, was associated with reduced happiness and relaxation and heightened frustration and boredom. Grounded in ACT and CVT, these findings position PF as a process‐based capacity that supports adolescents in managing evaluative stress and sustaining engagement. Integrating ACT‐informed practices into school‐based social–emotional learning may therefore strengthen emotion regulation, persistence and resilience in academic contexts.

## AUTHOR CONTRIBUTIONS

**Maryam Zarra‐Nezhad:** Conceptualization; visualization; project administration; writing – original draft; writing – review and editing. **Teemu Holopainen:** Methodology; software; formal analysis; writing – review and editing. **Pekka Räsänen:** Funding acquisition; conceptualization; methodology; writing – review and editing. **Mikko‐Jussi Laakso:** Funding acquisition; writing – review and editing. **Katarina Alanko:** Supervision; resources; writing – review and editing; investigation; conceptualization.

## CONFLICT OF INTEREST STATEMENT

The authors declare no conflicts of interest.

## Supporting information


Table S1–S4.

## Data Availability

The anonymized dataset from the DigiEva project is stored in a secure institutional repository. Due to research agreements with participating municipalities, access to the data is restricted. Data can be made available to qualified researchers upon reasonable request to the project coordinators, in accordance with institutional and data protection regulations.
