# Supplementary material for: Psychological flexibility and emotional trajectories during a semi‐high‐stakes mathematics exam: Integrating acceptance and commitment therapy with control‐value theory
Source: Br J Educ Psychol. 2026 Jul 21;96(4):1749–68. doi: 10.1111/bjep.70110 (PMC13752607; doi:10.1111/bjep.70110)
Supplement: Supplementary file 1 [file BJEP-96-1749-s001.docx]

**Supplementary Material**

The following supplementary material provides additional information supporting the main manuscript.

Table S1

Descriptive Statistics of CPFQ-9 Items

| Item | *n* | | | *M* | *SD* | *Mdn* | | *Trimmed* | | *MAD* | | Min | | Max | Range | Skewness | Kurtosis | | SE | |
| --- | --- | --- | --- | --- | --- | --- | --- | --- | --- | --- | --- | --- | --- | --- | --- | --- | --- | --- | --- | --- |
| 1. I try really hard every day. | | 692 | 2.72 | | 0.81 | 3 | 2.74 | | 0.74 | | 0 | | 4 | | 4 | -0.46 | 0.32 | 0.03 | |  |
| 2. If I fail, I try again right away to do better. | | 690 | 2.40 | | 0.89 | 2 | 2.39 | | 1.48 | | 0 | | 4 | | 4 | -0.18 | -0.33 | 0.03 | |  |
| 4. I notice my thoughts and feelings. | | 689 | 2.97 | | 0.88 | 3 | 3.05 | | 1.48 | | 0 | | 4 | | 4 | -0.80 | 0.68 | 0.03 | |  |
| 7. It’s OK to be scared. | | 686 | 2.37 | | 1.23 | 2 | 2.46 | | 1.48 | | 0 | | 4 | | 4 | -0.39 | -0.76 | 0.05 | |  |
| 8. If I think something that doesn’t mean it’s true. | | 687 | 2.56 | | 1.02 | 3 | 2.62 | | 1.48 | | 0 | | 4 | | 4 | -0.35 | -0.28 | 0.04 | |  |
| 11. It’s OK to feel mad. | | 683 | 2.88 | | 0.97 | 3 | 2.97 | | 1.48 | | 0 | | 4 | | 4 | -0.70 | 0.17 | 0.04 | |  |
| 12. If I do something bad, then I’m a bad person.* | | 685 | 2.60 | | 1.12 | 3 | 2.69 | | 1.48 | | 0 | | 4 | | 4 | -0.55 | -0.40 | 0.04 | |  |
| 13. I worry a lot about stuff I did or need to do.* | | 683 | 1.63 | | 1.14 | 2 | 1.57 | | 1.48 | | 0 | | 4 | | 4 | 0.33 | -0.62 | 0.04 | |  |
| 15. If I get angry, it means I messed up.* | | 677 | 2.60 | | 1.10 | 3 | 2.69 | | 1.48 | | 0 | | 4 | | 4 | -0.53 | -0.37 | 0.04 | |  |

*Note: n* = number of valid cases; *SD* = standard deviation; trimmed = trimmed mean (with trim defaulting to .1); *MAD* = median absolute deviation (from the median); skew = skewness; *SE* = standard error (of the mean); * = item was reverse scored; total sample (*N* = 694) included 338 girls and 356 boys from 8th–9th grades.

Table S2

Items and Factor Loadings for the CPFQ-9

| CPFQ Item | FA1 | | FA2 | | FA3 | | *h²* |
| --- | --- | --- | --- | --- | --- | --- | --- |
| 1. I try really hard every day. | 0.70 |  | |  | | 0.48 | |
| 2. If I fail, I try again right away to do better. | 0.75 |  | |  | | 0.56 | |
| 4. I notice my thoughts and feelings. | 0.56 |  | |  | | 0.32 | |
| 7. It’s OK to be scared. |  | 0.75 | |  | | 0.56 | |
| 8. If I think something that doesn’t mean it’s true. |  | 0.54 | |  | | 0.29 | |
| 11. It’s OK to feel mad. |  | 0.65 | |  | | 0.42 | |
| 12. If I do something bad, then I’m a bad person. |  |  | | 0.52 | | 0.28 | |
| 13. I worry a lot about stuff I did or need to do. |  |  | | 0.60 | | 0.36 | |
| 15. If I get angry, it means I messed up. |  |  | | 0.76 | | 0.59 | |

*Note.* FA1 = Committed Action and Awareness; FA2 = Acceptance and Defusion; FA3 = Fusion and Self-Judgment; *h²* = communality.

Table S3

Measurement Invariance Model Fit Indices

| Group | Model | χ² | *df* | CFI | ΔCFI | TLI | ΔTLI | SRMR | RMSEA | ΔRMSEA | 90% CI RMSEA |
| --- | --- | --- | --- | --- | --- | --- | --- | --- | --- | --- | --- |
| Total sample | Constrained model | 120.278*** | 24 | 0.917 |  | 0.875 |  | 0.067 | 0.076 |  | [0.063, 0.090] |
|  | Adjusted model^a^ | 93.618*** | 23 | 0.939 |  | 0.904 |  | 0.059 | 0.067 |  | [0.053, 0.081] |
| Gender | Configural | 90.336*** | 46 | 0.960 |  | 0.938 |  | 0.052 | 0.053 |  | [0.036, 0.069] |
|  | Metric | 109.513*** | 52 | 0.948 | 0.012 | 0.928 | 0.010 | 0.058 | 0.056 | 0.003 | [0.042, 0.071] |
|  | Scalar | 191.883*** | 58 | 0.880 | 0.068 | 0.850 | 0.078 | 0.070 | 0.082 | 0.026 | [0.069, 0.095] |
|  | Partial scalar^b^ | 120.464*** | 55 | 0.941 | 0.061 | 0.923 | 0.073 | 0.060 | 0.059 | 0.023 | [0.044, 0.073] |

*Note.* χ² = chi-square test; *df* = degrees of freedom; CFI = Comparative Fit Index; TLI = Tucker–Lewis Index; SRMR = standardized root mean square residual; RMSEA = root mean square error of approximation; CI = confidence interval.
ᵃ Residuals of Items 1 and 2 correlated.
ᵇ Intercepts of Items 1, 7, and 13 freed.

**Table S1A**

*Model Comparison for the Random Effects Structure of Happiness Trajectories*

| **Model** | **Random Structure** | **AIC** | **BIC** | **χ²** | **df** | ***p*** |
| --- | --- | --- | --- | --- | --- | --- |
| M0 | (1 \| ID) | 10,123.6 | 10,149.0 |  |  | — |
| M1 | (time linear \| ID) | 9,936.5 | 9,975.0 | 191.06 | 2 | **< 0.001** |

*Note.* Models were estimated using restricted maximum likelihood (REML) and compared via likelihood ratio tests (LRT). M0 = intercept-only random effects (baseline model); M1 = random intercept and random slope for linear time. AIC = Akaike Information Criterion; BIC = Bayesian Information Criterion; χ² = chi-square statistic from LRT; df = degrees of freedom for the LRT; ID = participant identifier. A significant χ² indicates that the more complex random structure provides a significantly better fit to the data. p-values for LRTs on variance components are conservative (boundary testing). — = not applicable.

**Table S2A**

Competing Model Comparisons for the Happiness Model

| **Model** | **Fixed Effects** | **k** | **AIC** | **BIC** | **logLik** | **Chi2** | **Ddf** | ***p*** |
| --- | --- | --- | --- | --- | --- | --- | --- | --- |
| *—* | *Constant across all models: gender + T10* | *—* | *—* | *—* | *—* | *—* | *—* | *—* |
| H1 | time linear + time quadratic + PF-Action | 10 | 9545.8 | 9609.9 | -4762.9 | — | — | — |
| H2 | + PI-Judge | 11 | 9542.5 | 9613 | -4760.3 | 5.29 | 1 | **0.021** |
| H3 | + PF-Accept | 12 | 9544.4 | 9621.3 | -4760.2 | 0.11 | 1 | 0.741 |
| H4 | + time quadratic × PF-Action | 12 | 9540.1 | 9617 | -4758.1 | 4.41 | 1 | **0.036** |
| H5 | + time linear × PF-Action | 13 | 9538.3 | 9621.6 | -4756.1 | 3.84 | 1 | 0.050* |

*Note.* All models included random intercepts and random slopes for linear time (time | ID), estimated with maximum likelihood. PF-Action = Committed Action Awareness; PI-Judge = Fusion with Self-Judgement; PF-Accept = Acceptance and Defusion; k = number of model parameters; AIC = Akaike Information Criterion; BIC = Bayesian Information Criterion; logLik = log-likelihood. H2 tested the addition of PI-Judge and was retained. H3 tested the addition of PF-Accept and was rejected. H5 tested whether time interaction with PF-Action had both linear and quadratic effect and produced a *borderline *p*-value. BIC favoured the simpler H4 with only quadratic interaction term. Therefore, H4 was selected as the final model.

**Table S3A**

*Original and Adjusted p-Values for Fixed Effects Predicting Happiness*

| **Predictor** | ***p*** | ***p_adj_*** |
| --- | --- | --- |
| (Intercept) | **< 0.001** | **< 0.001** |
| gender [girl] | **< 0.001** | **< 0.001** |
| T10 | **0.002** | **0.003** |
| time linear | **< 0.001** | **< 0.001** |
| time quadratic | **< 0.001** | **< 0.001** |
| PF-Action | **< 0.001** | **< 0.001** |
| PI-Judge | **0.022** | **0.025** |
| *time quadratic × PF-Action* | **0.036** | **0.036** |

*Note. p* = original *p*-value from the linear mixed model; padj = *p*-value adjusted for multiple comparisons using the Benjamini-Hochberg false discovery rate (FDR) procedure.

**Table S4A**

*Random Effects and Model Fit Statistics for the Final Linear Mixed Model for Happiness*

| **Parameter** | **Value** |
| --- | --- |
| *σ²* | 0.28 |
| *τ00 ID* | 0.67 |
| *τ11 ID × timeₗ* | 0.06 |
| *ρ01 ID* | 0.12 |
| ICC | 0.72 |
| N (ID) | 689 |
| Observations | 4474 |
| Marginal R² | 0.157 |
| Conditional R² | 0.766 |

*Note.* σ² = residual variance; τ00 = between-person variance in intercepts; τ11 = between-person variance in linear time slopes; ρ01 = correlation between random intercept and random slope; ICC = intraclass correlation coefficient, indicating the proportion of total variance attributable to between-person differences; N = number of participants; timeₗ = linear time predictor. Marginal R² reflects variance explained by fixed effects only; Conditional R² reflects variance explained by both fixed and random effects.

**Table S1B**

*Model Comparison for the Random Effects Structure of Relaxation Trajectories*

| **Model** | **Random Structure** | **AIC** | **BIC** | **χ²** | **df** | ***p*** |
| --- | --- | --- | --- | --- | --- | --- |
| M0 | (1 \| ID) | 11,097.0 | 11,123.0 |  |  | — |
| M1 | (time linear \| ID) | 10,948.0 | 10,986.0 | 153.52 | 2 | **< 0.001** |

*Note.* Models were estimated using restricted maximum likelihood (REML) and compared via likelihood ratio tests (LRT). M0 = intercept-only random effects (baseline model); M1 = random intercept and random slope for linear time. AIC = Akaike Information Criterion; BIC = Bayesian Information Criterion; χ² = chi-square statistic from LRT; df = degrees of freedom for the LRT; ID = participant identifier. A significant χ² indicates that the more complex random structure provides a significantly better fit to the data. p-values for LRTs on variance components are conservative (boundary testing). — = not applicable.

**Table S2B**

*Competing Model Comparisons for the Relaxation Model*

| **Model** | | **Fixed Effects** | **k** | **AIC** | **BIC** | **logLik** | **Chi2** | **Ddf** | ***p*** |
| --- | --- | --- | --- | --- | --- | --- | --- | --- | --- |
| — | Constant across all models: gender + T10 + PI-Judge + time linear + time quadratic | | — | — | — | — | — | — | — |
| R1 | base model | | 10 | 10732 | 10796 | -5355.9 | — | — | — |
| R2 | + PF-Accept | | 11 | 10722 | 10792 | -5349.8 | 12.05 | 1 | **< 0.001** |
| R3 | + time quadratic × PF-Accept | | 12 | 10718 | 10795 | -5347.1 | 5.47 | 1 | **0.019** |
| R4 | time quadratic × PF-Accept × gender | | 15 | 10724 | 10820 | -5347 | 0.15 | 3 | 0.986 |
| R5 | + time linear × PF-Accept | | 13 | 10720 | 10803 | -5346.8 | 0.53 | 1 | 0.467 |
|  | | | | | | | | | |

*Note.* All models included random intercepts and random slopes for linear time (time | ID), estimated with maximum likelihood. PI-Judge = Fusion with Self-Judgement; PF-Accept = Acceptance and Defusion; k = number of model parameters; AIC = Akaike Information Criterion; BIC = Bayesian Information Criterion; logLik = log-likelihood. Model R2 tested the addition of PF-Accept and was retained. R3 tested the interaction of PF-Accept with quadratic time. R4 tested whether the interaction extended to a three-way interaction with gender and was rejected. R5 tested whether time interaction with PF-Accept had also a linear component and was rejected (against R3). R3 was selected as the final model.

**Table S3B**

*Original and Adjusted p-Values for Fixed Effects Predicting Relaxation*

| **Predictor** | ***p*** | ***p_adj_*** |
| --- | --- | --- |
| (Intercept) | **< 0.001** | **< 0.001** |
| gender [girl] | **< 0.001** | **< 0.001** |
| T10 | **< 0.001** | **< 0.001** |
| time linear | **< 0.001** | **< 0.001** |
| time quadratic | **< 0.001** | **< 0.001** |
| PF-Accept | **0.009** | **0.010** |
| PI-Judge | **< 0.001** | **< 0.001** |
| time quadratic *×* PF-Accept | **0.019** | **0.019** |

*Note. p* = original *p*-value from the linear mixed model; padj = *p*-value adjusted for multiple comparisons using the Benjamini-Hochberg false discovery rate (FDR) procedure.

**Table S4B**

*Random Effects and Model Fit Statistics for the Relaxation Model*

| **Parameter** | **Value** |
| --- | --- |
| *σ²* | 0.39 |
| *τ00 ID* | 0.73 |
| *τ11 ID × timeₗ* | 0.06 |
| *ρ01 ID* | 0.19 |
| ICC | 0.67 |
| N (ID) | 689 |
| Observations | 4466 |
| Marginal R² | 0.141 |
| Conditional R² | 0.717 |

*Note.* σ² = residual variance; τ00 = between-person variance in intercepts; τ11 = between-person variance in linear time slopes; ρ01 = correlation between random intercept and random slope; ICC = intraclass correlation coefficient, indicating the proportion of total variance attributable to between-person differences; N = number of participants; timeₗ = linear time predictor. Marginal R² reflects variance explained by fixed effects only; Conditional R² reflects variance explained by both fixed and random effects.

**Table S1C**

*Model Comparison for the Random Effects Structure of Frustration Trajectories*

| **Model** | **Random Structure** | **AIC** | **BIC** | **χ²** | **df** | ***p*** |
| --- | --- | --- | --- | --- | --- | --- |
| M0 | (1 \| ID) | 13,017.0 | 13,042.0 |  |  | — |
| M1 | (time linear \| ID) | 12,800.0 | 12,838.0 | 221.19 | 2 | **< 0.001** |

*Note.* Models were estimated using restricted maximum likelihood (REML) and compared via likelihood ratio tests (LRT). M0 = intercept-only random effects (baseline model); M1 = random intercept and random slope for linear time. AIC = Akaike Information Criterion; BIC = Bayesian Information Criterion; χ² = chi-square statistic from LRT; df = degrees of freedom for the LRT; ID = participant identifier. A significant χ² indicates that the more complex random structure provides a significantly better fit to the data. p-values for LRTs on variance components are conservative (boundary testing). — = not applicable.

**Table S2C**

*Competing Model Comparisons for the Frustration Model*

| **Model** | **Fixed Effects** | **k** | **AIC** | **BIC** | **logLik** | **Chi2** | **Ddf** | ***p*** |
| --- | --- | --- | --- | --- | --- | --- | --- | --- |
| *—* | *Constant across all models: gender + T10 + PI-Judge + PF-Accept + PF-Action + time linear + time quadratic* | *—* | *—* | *—* | *—* | *—* | *—* | *—* |
| F1 | base model | 12 | 12744 | 12821 | -6360 | — | — | — |
| F2 | + time linear × PF-Action | 13 | 12739 | 12822 | -6356.6 | 6.65 | 1 | **0.010** |
| F3 | + time quadratic × PF-Action | 14 | 12734 | 12824 | -6353.3 | 6.71 | 1 | **0.010** |
| F4 | + time quadratic × PF-Accept | 15 | 12729 | 12825 | -6349.6 | 7.37 | 1 | **0.007** |
| F5 | + time linear × PF-Accept | 16 | 12728 | 12831 | -6348.3 | 2.65 | 1 | 0.103 |

*Note.* All models included random intercepts and random slopes for linear time (time | ID), estimated with maximum likelihood. PF-Action = Committed Action Awareness; PI-Judge = Fusion with Self-Judgement; PF-Accept = Acceptance and Defusion; k = number of model parameters; AIC = Akaike Information Criterion; BIC = Bayesian Information Criterion; logLik = log-likelihood. Model F2 tested the interaction of linear time with PF-Action and was retained. F3 tested the curvature of the interaction as quadratic time with PF-Action term, which was retained. F4 tested adding interaction of quadratic time with PF-Accept F4. F5 tested whether there was also a linear interaction effect of time and PF-Accept and was rejected. F4 was selected as the final model.

**Table S3C**

*Original and Adjusted p-Values for Fixed Effects Predicting Frustration*

| **Predictor** | ***p*** | ***p_adj_*** |
| --- | --- | --- |
| (Intercept) | **< 0.001** | **<0.0** |
| gender [girl] | 0.359 | 0.494 |
| T10 | 0.681 | 0.751 |
| time linear | **< 0.001** | **< 0.001** |
| time quadratic | 0.541 | 0.662 |
| PF-Accept | 0.271 | 0.426 |
| PF-Action | 0.911 | 0.911 |
| PI-Judge | **< 0.001** | **< 0.001** |
| time linear × PF-Action | **0.012** | **0.022** |
| time quadratic × PF-Action | **< 0.001** | **0.001** |
| time quadratic × PF-Accept | **0.007** | **0.015** |

*Note. p* = original p-value from the linear mixed model; padj = *p*-value adjusted for multiple comparisons using the Benjamini-Hochberg false discovery rate (FDR) procedure.

**Table S4C**

Random Effect Model Fit Statistics for the Frustration Model

| **Parameter** | **Value** |
| --- | --- |
| *σ²* | 0.60 |
| *τ00 ID* | 1.35 |
| *τ11 ID × timeₗ* | 0.11 |
| *ρ01 ID* | 0.39 |
| ICC | 0.71 |
| N (ID) | 689 |
| Observations | 4457 |
| Marginal R² | 0.044 |
| Conditional R² | 0.718 |

*Note.* σ² = residual variance; τ00 = between-person variance in intercepts; τ11 = between-person variance in linear time slopes; ρ01 = correlation between random intercept and random slope; ICC = intraclass correlation coefficient, indicating the proportion of total variance attributable to between-person differences; N = number of participants; timeₗ = linear time predictor. Marginal R² reflects variance explained by fixed effects only; Conditional R² reflects variance explained by both fixed and random effects.

**Table S1D**

*Model Comparison of the Random Effects Structure of Boredom Trajectories*

| **Model** | **Random Structure** | **AIC** | **BIC** | **χ²** | **df** | ***p*** |
| --- | --- | --- | --- | --- | --- | --- |
| M0 | (1 \| ID) | 13,573.0 | 13,599.0 |  |  | — |
| M1 | (time linear \| ID) | 13,379.0 | 13,417.0 | 198.4 | 2 | **< 0.001** |

*Note.* Models were estimated using restricted maximum likelihood (REML) and compared via likelihood ratio tests (LRT). M0 = intercept-only random effects (baseline model); M1 = random intercept and random slope for linear time. AIC = Akaike Information Criterion; BIC = Bayesian Information Criterion; χ² = chi-square statistic from LRT; df = degrees of freedom for the LRT; ID = participant identifier. A significant χ² indicates that the more complex random structure provides a significantly better fit to the data. p-values for LRTs on variance components are conservative (boundary testing). — = not applicable.

**Table S2D**

*Competing Model Comparisons for the Boredom Model*

| **Model** | **Fixed Effects** | **k** | **AIC** | **BIC** | **logLik** | **Chi2** | **Ddf** | ***p*** |
| --- | --- | --- | --- | --- | --- | --- | --- | --- |
| *—* | *Constant across all models: gender + T10 + PI-Judge + time linear + time quadratic* | *—* | *—* | *—* | *—* | *—* | *—* | *—* |
| B1 | base model | 10 | 13323 | 13387 | -6651.3 | — | — | — |
| B2 | + PF-Accept | 11 | 13323 | 13393 | -6650.4 | 1.79 | 1 | 0.181 |
| B3 | + gender × PI-Judge | 11 | 13320 | 13390 | -6648.9 | 4.82 | 1 | **0.028** |
| B4 | + gender × PI-Judge × time linear | 14 | 13320 | 13409 | -6645.8 | 6.17 | 3 | 0.103 |

*Note.* All models included random intercepts and random slopes for linear time (time | ID), estimated with maximum likelihood. PF-Action = Committed Action Awareness; PI-Judge = Fusion with Self-Judgement; PF-Accept = Acceptance and Defusion; k = number of model parameters; AIC = Akaike Information Criterion; BIC = Bayesian Information Criterion; logLik = log-likelihood. Model B2 tested the addition of PF-Accept and was rejected. B3 tested the addition of interaction of gender with PI-Judge. B4 tested whether the gender x PI-Judge interaction extended to a three-way interaction with linear time against B3 and was rejected. B3 was selected as the final model.

**Table S3D**

*Original and Adjusted p-Values for Fixed Effects Predicting Boredom*

| **Predictor** | ***p*** | ***p_adj_*** |
| --- | --- | --- |
| (Intercept) | **< 0.001** | **<0.001** |
| gender [girl] | **0.001** | **0.002** |
| T10 | **< 0.001** | **< 0.001** |
| time linear | **< 0.001** | **< 0.001** |
| time quadratic | **< 0.001** | **< 0.001** |
| PI-Judge | **< 0.001** | **< 0.001** |
| gender [girl] × PI-Judge | **0.029** | **0.028** |

*Note. p* = original p-value from the linear mixed model; padj = *p*-value adjusted for multiple comparisons using the Benjamini-Hochberg false discovery rate (FDR) procedure.

**Table S4D**

| **Parameter** | **Value** |
| --- | --- |
| *σ²* | 0.69 |
| *τ00 ID* | 1.33 |
| *τ11 ID × timeₗ* | 0.12 |
| *ρ01 ID* | 0.34 |
| ICC | 0.67 |
| N (ID) | 689 |
| Observations | 4480 |
| Marginal R² | 0.030 |
| Conditional R² | 0.684 |

*Random Effects and Model Fit Statistics for the Boredom Model*

*Note.* σ² = residual variance; τ00 = between-person variance in intercepts; τ11 = between-person variance in linear time slopes; ρ01 = correlation between random intercept and random slope; ICC = intraclass correlation coefficient, indicating the proportion of total variance attributable to between-person differences; N = number of participants; timeₗ = linear time predictor. Marginal R² reflects variance explained by fixed effects only; Conditional R² reflects variance explained by both fixed and random effects.
